# Global DNA Methylation in Dental Implant Failure Due to Peri-Implantitis: An Exploratory Clinical Pilot Study

**DOI:** 10.3390/ijerph19021020

**Published:** 2022-01-17

**Authors:** Ismael Khouly, Simon Pardiñas López, Silvia María Díaz Prado, Luca Ferrantino, Josephine Kalm, Lena Larsson, Farah Asa’ad

**Affiliations:** 1 Department of Oral and Maxillofacial Surgery, College of Dentistry, New York University, New York, NY 10010, USA; 2 Periodontology and Oral Surgery, Clínica Médico Dental Pardiñas, Real 66, 3°, 15003 A Coruña, Spain; s.pardinas@clinicapardinas.com; 3 Institute of Biomedical Research of A Coruña (INIBIC), Galician Health Service (SERGAS), University Hospital Complex A Coruña (CHUAC), 15006 A Coruña, Spain; silvia.ma.diaz.prado@sergas.es; 4 Centro de Investigaciones Científicas Avanzadas (CICA), University of A Coruña, Rúa As Casballeiras, 15071 A Coruña, Spain; 5 Cell Therapy and Regenerative Medicine Group, Department of Physiotherapy, Medicine and Biomedical Sciences, Faculty of Health Sciences, University of A Coruña (UDC), 15006 A Coruña, Spain; 6 Centro de Investigación Biomédica en Red (CIBER) de Bioingeniería, Biomateriales y Nanomedicina (CIBER-BBN), 28029 Madrid, Spain; 7 Department of Biomedical, Surgical, and Dental Sciences, University of Milan, 20122 Milan, Italy; luca.ferrantino@gmail.com; 8 Department of Aesthetic Dentistry, Istituto Stomatologico Italiano, 20122 Milan, Italy; 9 Department of Periodontology, Institute of Odontology, The Sahlgrenska Academy at University of Gothenburg, SE-405 30 Göteborg, Sweden; josephine.kalm@gu.se (J.K.); lena.larsson@odontologi.gu.se (L.L.); 10 Department of Biomaterials, Institute of Clinical Sciences, The Sahlgrenska Academy at University of Gothenburg, SE-405 30 Göteborg, Sweden; farah.asaad@gu.se; 11 Department of Oral Biochemistry, Institute of Odontology, The Sahlgrenska Academy at University of Gothenburg, SE-405 30 Göteborg, Sweden

**Keywords:** DNA methylation, epigenomics, dental implantation, peri-implantitis, periodontium

## Abstract

Background: Peri-implantitis (PIT) is highly prevalent in patients with dental implants and is a challenging condition to treat due to the limited outcomes reported for non-surgical and surgical therapies. Therefore, epigenetic therapeutics might be of key importance to treat PIT. However, developing epigenetic therapeutics is based on understanding the relationship between epigenetics and disease. To date, there is still scarce knowledge about the relationship between epigenetic modifications and PIT, which warrants further investigations. Aim: The purpose of this study was to evaluate the level of global DNA methylation associated with implant failure (IF) due to PIT compared to periodontally healthy (PH) patients. Material and Methods: A total of 20 participants were initially enrolled in this pilot, exploratory, single-blinded, cross-sectional clinical human study in two groups: 10 in the PH group and 10 in the IF group. In the participants who have completed the study, gingival tissue and bone samples were harvested from each participant and were used to perform global DNA methylation analysis. The percentage of global DNA methylation (5-mC%) was compared (1) between groups (PH and IF); (2) between the subgroups of gingival tissue and bone separately; (3) in the whole sample, comparing gingival tissue and bone; (4) within groups, comparing gingival tissue and bone. Demographic, periodontal, and peri-implant measurements as well as periodontal staging, were also recorded. All statistical comparisons were made at the 0.05 significance level. Results: Out of the initially enrolled 20 patients, only 19 completed the study and, thus, were included in the final analysis; 10 patients in the PH group and 9 patients in the IF group, contributing to a total of 38 samples. One patient from the IF group was excluded from the study due to systemic disease. The mean implant survival time was 10.8 years (2.17–15.25 years). Intergroup comparison, stratified by group, indicated a similar 5-mC% between the PH and IF groups in both gingival tissue and bone (*p* = 0.599), only in bone (*p* = 0.414), and only in gingival tissue (*p* = 0.744). Intragroup comparison, stratified by the type of sample, indicated a significantly higher 5-mC% in gingival tissue samples compared to bone in both the PH and IF groups (*p* = 0.001), in the PH group (*p* = 0.019), and in the IF group (*p* = 0.009). Conclusions: Within the limitations of this study, higher global DNA methylation levels were found in gingival tissue samples compared to bone, regardless of the study groups. However, similar global DNA methylation levels were observed overall between the IF and PH groups. Yet, differences in the global DNA methylation levels between gingival tissues and bone, regardless of the study group, could reflect a different epigenetic response between various tissues within the same microenvironment. Further studies are necessary to elucidate the present findings and to evaluate the role of epigenetic modifications in IF due to PIT.

## 1. Introduction

Despite the well-established influence of epigenetic modifications in cancer and inflammatory diseases [1,2,3,4,5], little is known about these modifications in the context of oral health [6]. One of the major epigenetic mechanisms is DNA methylation, which refers to chemical alterations of the DNA through the activity of specific enzymes, resulting in the modulation of gene expression without changing the DNA sequence [7].

Low DNA methylation levels within the CpG islands of a gene promoter are associated with transcriptionally active genes, in contrast to high DNA methylation levels [7,8].

Recent studies on DNA methylation have shown differential DNA methylation of genes related to the immune response in patients with periodontitis compared to those with a healthy periodontium [9,10,11,12]. However, there is scarce evidence on the association between DNA methylation and peri-implantitis (PIT).

Similar to periodontitis, PIT represents a disturbance in the interactions between the microbial and the host immunological responses, which are influenced in part by epigenetics [13,14,15]. As such, epigenetic modifications might play key roles in the initiation and progression of PIT, resulting ultimately in implant failure (IF), and therefore, they could serve as diagnostic biomarkers in the future to indicate the risk of disease, and also as prognostic biomarkers of PIT progression and subsequent IF [16].

Since the increased placement of dental implants in everyday clinical practice increases the possibility of concomitant occurrence of PIT, there is an urgent need to understand the biological events that underlie the risk for IF due to PIT, such as epigenetics. In fact, understanding the altered epigenetic state of cells could help in the development of epigenetic therapies [4], which might have a major clinical impact on the treatment outcomes of PIT, not only due to the high prevalence of PIT [17] but also due to the limited treatment efficacy of PIT with the conventional treatment methods, whether the non-surgical [18,19] or surgical approaches [20,21,22]. Nonetheless, realizing the promise of epigenetic therapies will require a deeper understanding of how epigenetic mechanisms induce the progression of PIT, resulting ultimately in IF.

In this pilot, exploratory, single-blinded, cross-sectional study, we seek to characterize the global DNA methylation in gingival tissue and bone samples from patients with IF due to PIT in comparison to the global DNA methylation in gingival tissue and bone samples from periodontally healthy (PH) patients. Herein, we hypothesized that global DNA methylation levels, expressed in terms of global percentage of CpG sites in genomic DNA, are different between the IF and PH groups. Furthermore, we hypothesized that global DNA methylation levels are different between gingival tissues and bone. 

## 2. Material and Methods

### 2.1. Study Design

This study was conducted as a pilot, exploratory, single-blinded, cross-sectional clinical human study. From the pool of patients attending a private dental practice in Spain, 20 participants were initially enrolled in 2 groups: 10 PH patients and 10 patients diagnosed with IF. Patients who completed the study contributed to two types of samples; gingival tissue and bone.

Patient enrollment took place in the period between January 2019 and December 2019.

### 2.2. Setting

Patient screening, enrollment, study procedures, surgical interventions, data collection, and sample harvesting were conducted at a private dental practice in Spain (Clinica Médico Dental Pardiñas, A Coruña). All patients provided written consent prior to any of the study procedures.

The harvested samples were stored at the Biobank of A Coruña, Xerencia Xestión Integrada de A Coruña, University Hospital Center of A Coruña, Spain (registration number: 2017/104) and the DNA extraction from samples was done at Instituto de Investigación Biomédica de A Coruña (INIBIC), University of A Coruña, Spain.

The epigenetic analysis was performed at the University of Gothenburg, Göteborg, Sweden.

### 2.3. Ethical Approval and Study Registration

This study was performed following the ethical principles outlined in the declaration of Helsinki and the International Conference on Harmonisation (ICH)-Good Clinical Practice (GCP) guidelines and is in accordance with the STROBE guidelines (Strengthening the Reporting of Observational Studies in Epidemiology) [23].

This study was approved by the A Coruña-Ferrol Territorial Research Ethics Committee (number: 2018/594) and is registered at ClinicalTrials.gov (Identifier: NCT04421066).

### 2.4. Eligibility Criteria for Study Participants

#### 2.4.1. General Inclusion Criteria

Study participants fit the following inclusion criteria:

1. Patients of 20–90 years of age;
2. Patients who read, understood, and signed the informed consent form;
3. Non-smokers or ex-smokers who had quit smoking for at least one year prior to enrollment in the study.

#### 2.4.2. Inclusion of Samples from PH Patients

In addition to the criteria listed in the section (General Inclusion Criteria), samples from PH patients were included in the study if the patient had healthy gingiva with an intact periodontium, without a history of periodontitis (based on the 2017 World Workshop on the Classification of Periodontal and Peri-Implant Diseases and Conditions) [24,25,26].

#### 2.4.3. Inclusion of Samples from Patients with IF

In addition to the criteria listed in the section (General Inclusion Criteria), samples from IF patients were included in the study if the patient had at least one implant-supported prosthesis/es, which was in function for at least 12 months but required removal due to progressive PIT (based on the 2017 World Workshop on the Classification of Periodontal and Peri-Implant Diseases and Conditions) [26] with peri-implant crestal bone loss greater than 50% of the implant fixture length [27].

#### 2.4.4. General Exclusion Criteria

Study participants who fit any of the following criteria were excluded from the study:

1. Pregnant or lactating patients at the time of sample harvesting;
2. Non-compliant patients;
3. Patients who reported the use of antibiotics and/or non-steroidal anti-inflammatory drugs (NSAIDS), for at least 1 month before sample harvesting. However, the use of low-dose aspirin (≤81 mg/day) for prophylaxis was allowed;
4. Patients with a history of receiving intravenous or subcutaneous anti-resorptive agents associated with osteonecrosis of the jaw, such as bisphosphonates;
5. Patients with mucosal diseases (e.g., erosive lichen planus) in the localized area around the sample site;
6. Patients with systemic diseases, including autoimmune conditions that would preclude the analysis (e.g., diabetes mellitus, bleeding disorders, bone metabolism disorders, and systemic lupus erythematosus);
7. Patients with a history of local irradiation therapy in the head/neck area;
8. Patients who demonstrated the following upon dental examination: acute necrotizing ulcerative gingivitis, poor oral hygiene, untreated endodontic lesions, gross tooth decay, or periodontal disease in the area adjacent to the sample site.

#### 2.4.5. Exclusion of Samples from PH Patients

Samples from PH patients were excluded from the study if bleeding on probing (BOP), root fragments, pericoronitis, endo-perio lesions, gross tooth decay, or dental abscess were present at the site intended for biopsy.

#### 2.4.6. Exclusion of Samples from Patients with IF

Samples from IF patients were excluded from the study if the IF was due to inadequate implant position (i.e., prosthetically driven), implant mobility, excess cement, or if the patient had implants previously treated surgically for PIT and is using antibiotics as part of the treatment.

### 2.5. Outcomes of Interest

The primary outcome of the study was the global DNA methylation levels in gingival tissues and bone.

### 2.6. Study Procedures

All patients were seen by the same experienced clinician (S.P.L). After signing the informed consent, patients were assessed for initial eligibility to be enrolled in the study. This study enclosed one visit only for all the participating patients.

The following assessments and procedures were performed by the same experienced clinician (S.P.L): medical and dental history, demographics (e.g., gender, age, race, ethnicity, and history of tobacco use), intraoral full-mouth radiographic series, review the inclusion/ exclusion criteria if necessary, oral examination, oral hygiene assessment, and clinical parameters, including probing depth (PD), gingival recession (GR), clinical attachment level (CAL), and bleeding on probing (BOP) (Supplementary File S1). Periodontal staging, based on the 2017 World Workshop on the Classification of Periodontal and Peri-Implant Diseases and Conditions [24,25], was also recorded. Clinical measurements of PD, CAL, and BOP were recorded at six points around each tooth and dental implant during the study visit by the same experienced clinician (S.P.L). For IF patients, keratinized tissue height (KT) was also measured, and peri-apical intraoral radiographs of the failed dental implants were taken in order to measure crestal bone loss (BL) (Supplementary File S1).

### 2.7. Samples

Two types of samples were harvested as part of this study: gingival tissue (of approximately 3 mm diameter) and bone from both PH and IF patients.

For the PH group, samples were collected from patients who underwent a surgical extraction of wisdom teeth. The samples, which included a section of the gingival tissue and bone removed at that extraction site, were taken for research purposes.

For the IF group only, samples were collected from patients who underwent surgical removal of an implant due to PIT. Implant removal was performed using a trephine bur under saline irrigation, allowing to obtain fragments of bone attached to the implant surface and gingival tissue at the IF site. The samples were taken for research purposes.

### 2.8. Extraction of DNA from Samples

Tissue samples were stored in Allprotect Tissue Reagent (Qiagen) at 4 °C until processing. For tissue homogenization, Allprotect Tissue Reagent was removed, and the samples were washed twice with PBS (Thermo Fisher Scientific, Madrid, Spain), placed in 2 mL tubes (Eppendorf Ibérica S.L.U., Madrid, Spain) with zirconium oxide grinding balls (Retsch, Haan, Germany) and frozen in liquid nitrogen. A Mixer Mill MM200 (Retsch) was used to homogenize the samples. Cycles of 25 Hz frequency and 5 min duration were performed until complete homogenization of the samples (about 8 cycles for bone and 6 for gingiva), re-freezing the samples between cycles.

Genomic DNA was isolated from the homogenates using AllPrep DNA/RNA/miRNA Universal Kit (Qiagen), precipitated in isopropanol (Sigma-Aldrich Química S.A., Madrid, Spain), washed in 70° ethanol (Sigma-Aldrich Química S.A.), left to dry, and then resuspended in sterile water spectrophotometer (Thermo Fisher Scientific). DNA quantity and quality were assessed using a NanoDropTM spectrophotometer (Thermo Fisher Scientific) with wavelengths of 260 nm (for quantification), 230 nm, and 260 nm (for quality and purity).

### 2.9. DNA Methylation Assay

Differences in the global DNA methylation levels between gingival tissue and bone were analyzed in the PH and IF groups. Global DNA methylation analysis was performed using a MethylFlash Methylated DNA Quantification kit (Epigentek, Farmingdale, NY, USA), according to the manufacturer’s instructions. Briefly, 20 ng of input DNA of 260/280 ratio > 1.6 was assayed in duplicates, along with positive and negative controls, in strip wells specifically treated to have high DNA affinity.

The methylated fraction of DNA was detected using capture and detection antibodies and quantified colorimetrically by reading the absorbance in a microplate spectrophotometer at 450 nm within 5–15 min. The percentage of methylated DNA was relatively quantified using a formula provided by the manufacturer, expressed as a percentage of 5-mC/sample (5-mC%) [28,29].

### 2.10. Statistical Analysis

Data were collected and checked for entry errors using a Microsoft Excel sheet. Continuous variables were tested for normal distribution with Shapiro-Wilk’s method and then summarized with mean and standard deviation (SD) or with median and interquartile range (IQR) in case of non-normality. Proportion was used to describe categorical variables. The baseline demographic and clinical characteristics were reported for both groups and compared. The percentage of global DNA methylation was compared (1) between groups (PH and IF); (2) between the subgroups of gingival tissue and bone separately; (3) in the whole sample, comparing gingival tissue and bone; (4) within groups, comparing gingival tissue and bone. The null hypothesis of no difference between groups was used for statistical tests. Continuous variable comparison was performed using the unpaired Student’s t-test (or Mann-Whitney U test for non-normal data), while categorical variables were compared using Fisher’s Exact test.

All statistical comparisons were made at the 0.05 significance level using R software (R Foundation for Statistical Computing, Vienna, Austria).

## 3. Results

Out of the initially enrolled 20 patients, only 19 completed the study and, thus, were included in the final analysis; 10 patients in the PH group and 9 patients in the IF group, contributing to a total of 38 samples. One patient from the IF group was excluded from the study; after initial enrollment, the participant reported a diagnosis with systemic disease, which was part of the exclusion criteria.

The PH group contributed to a total of 20 samples (10 gingival tissue and 10 bone samples) from third molar extraction sites, with a total of 6 maxillary third molars and 4 mandibular third molars. A total of 7 third molars were fully erupted, and 3 third molars were partially erupted.

The IF group contributed to a total of 18 samples (9 gingival tissue and 9 bone samples) from 9 failed implants. The implants were in function for a mean of 10.8 years (2.17–15.25 years). The implant diameter varied between 3.0 and 5.0 mm (mean = 3.95 mm), and implant length varied between 8.5 and 13 mm (mean = 10.1mm). The implant location was as follows: 4 mandibular molars, 2 mandibular premolars, 2 maxillary molars and 1 maxillary premolar. All failed implants were nonmobile prior to dental implant removal. Supplementary Table S1 shows further information related to the characteristics of the removed implants.

### 3.1. Demographics

A total of 19 patients completed the study, 13 females (68.4%) and 6 males (31.6%). The mean patient age was 30.90 ± 6.76 years in the PH group and 63.33 ± 11.84 years in the IF group. All patients were of Caucasian origin. Table 1 shows further information regarding group comparison at baseline. Table 2 displays the baseline clinical parameters around dental implants that were removed due to IF.

### 3.2. Global DNA Methylation (5-mC)

Intergroup comparison, stratified by group, indicated similar 5-mC% between the PH and IF groups when bone and gingival tissue were analyzed (2.48 [1.42, 5.40] vs. 2.80 [1.98, 4.86], respectively, *p* = 0.599), in only bone (1.64 [0.43, 2.76] 2.08 [1.73, 2.63], respectively, *p* = 0.414), and only gingival tissue (4.86 [2.33, 11.38] 5.01 [2.97, 10.50] ,respectively, *p* = 0.744) (Table 3).

Intragroup comparison stratified by the type of the sample indicated significantly higher 5-mC% in gingival tissue compared to bone when both PH and IF groups were analyzed (1.76 [1.17, 2.85] vs. 4.89 [2.60, 11.70], respectively, *p* = 0.001), in only PH (1.64 [0.43, 2.76] 4.86 [2.33, 11.38], respectively, *p* = 0.019), and only IF (2.08 [1.73, 2.63] 5.01 [2.97, 10.50], respectively, *p* = 0.009) (Table 4).

## 4. Discussion

### 4.1. Main Findings

In recent years, various studies have emerged focusing on the regulatory role of epigenetics in the inflammatory response, including periodontal and systemic diseases [9,10,11,12]. However, studies investigating the role of different epigenetic mechanisms, including the global DNA methylation, in peri-implant diseases are still scarce.

To the best of the authors’ knowledge, the present study is the first to evaluate the global DNA methylation levels of bone and gingival tissue around dental implants that have failed due to PIT.

The present study evaluated the global DNA methylation in IF due to PIT compared to PH patients. Although the results showed similar global DNA methylation levels between the IF and PH groups, higher global DNA methylation levels were found in gingival tissues compared to bone, regardless of the study group. In fact, these differences in the global DNA methylation levels between gingival tissues and bone could reflect a different epigenetic response between various tissues within the same microenvironment. Moreover, these differences could also reflect a more active gene expression in bone compared to gingival tissue samples. However, future studies on gingival tissues and bone from PIT lesions and IF are still needed in order to evaluate the DNA methylation of a specific panel of cytokine and inflammation-related genes to elucidate which pro- and anti-inflammatory genes are transcriptionally active or inactive in PIT and IF.

### 4.2. Agreement and Disagreement with Existing Evidence

To date, there is no available evidence on the relationship between global DNA methylation and IF due to PIT, with the available evidence being scarce and confined to the relationship between global DNA methylation and PIT, with only one published clinical study available to date [30]. However, this study assessed the global DNA methylation levels from peri-implant crevicular fluid (PICF) of 21 PIT cases and 24 healthy implants, instead of gingival tissue and bone. Furthermore, the presence of titanium particles was quantified in the PICF. The results of this study showed that PIT cases displayed significantly increased global DNA methylation levels in PICF [30]. Moreover, adjustment for the smoking status further strengthened the association between PIT and the increased global DNA methylation levels. Interestingly, PICF samples with higher quantities of titanium particles displayed significantly higher global DNA methylation levels, irrespective of the PIT status, suggesting that DNA methylation might be, in fact, affected by the dissolution of titanium particles. However, it must be highlighted that further in-depth investigation is required to conclude if the nature of the association between global DNA methylation and smoking/titanium particles is causal [30].

Despite these interesting findings by Daubert and co-workers [30], it is difficult to compare those findings with the results of the present study due to the differences in the study design as the present study investigated the global DNA methylation in IF due to PIT, while Daubert and colleagues [30] investigated the global DNA methylation in PIT, without stating the severity of the peri-implant disease. Also, different forms of tissue samples were used; the present study assessed the global DNA methylation in gingival tissue and bone samples, while Daubert et al. (2019) assessed the global DNA methylation in PICF [30]. It is important to highlight that DNA methylation is highly cell- and tissue- type-specific [31,32,33,34,35], which reflects tissue-specific functions [36].

Moreover, different healthy control groups were enrolled in both studies; the present study assessed the global DNA methylation in gingival tissue and bone samples from healthy tissues around natural teeth, while Daubert et al. (2019) utilized PICF from healthy dental implants as controls [30]. Importantly, the present study excluded smokers, while Daubert et al. (2019) [30] have adjusted for smoking as a confounding factor in relation to the global DNA methylation levels. It must be noted that this is an important factor to consider since smoking is a well-known factor to influence epigenetic mechanisms.

### 4.3. Study Limitations

The present study possesses a few limitations that do not allow for the generalization of the study findings, such as the small sample size and the negligible statistical differences between the study groups. Ideally, the healthy group should be dental implants without PIT, and all the study groups should have the same type of dental implant with the same surface. However, harvesting both gingival tissue and bone samples from healthy implants would present ethical concerns. Therefore, the tissues which are the closest in characteristics to the tissues around the dental implants are the tissues around natural teeth. Furthermore, having PH patients as the healthy group further explains the statistically significant difference in the age between both study groups, with a higher age average in the IF group compared to the PH group. However, it must be noted that age difference between groups can be a common finding in such type of investigations; in a recent study that analyzed genes with differential DNA methylation and messenger (mRNA) expression between PIT and periodontitis among smokers and non-smokers, Cho et al. (2020) [37] reported that the average patient age was significantly higher in the non-smoking PIT group, compared to the non-smoking periodontitis group. This could be explained by the fact that it is already a difficult task to recruit patients with PIT into a study, and thus, having their age match patients in other groups within the same study might be a challenge.

Another limitation of the present study might be that only global DNA methylation levels were evaluated between the study groups and samples and not the DNA methylation of a specific gene panel. However, the statistically significant difference of the global DNA methylation levels between different sample types, regardless of the study group, is of importance in paving the way for future research on the role of DNA methylation of a specific gene panel in IF due to PIT, which would increase our knowledge on the pathogenesis of PIT.

### 4.4. Future Research and Recommendations

Future studies with a larger sample size are needed in order to clarify whether the present findings are authentic or merely a random variation. In addition, future longitudinal studies, preferably with a split-mouth design, could be performed to evaluate any changes in the global DNA methylation levels before and after treatment. Other epigenetic mechanisms, such as the DNA methylation of a specific gene panel and the differential expression of microRNAs and histone modifications, must be evaluated in different sample types (i.e., gingival tissues, bone, and PICF) in IF due to PIT compared to PH patients, and also to healthy dental implants. Furthermore, due to the mixed cell population in biopsies from gingival tissues, it is highly recommended to use laser microdissection as previously described in literature to microdissect gingival tissues, which would enable the assessment of epigenetic patterns of the epithelial component and the connective tissue component separately [38], and thus, overcome the challenge of cellular heterogeneity of gingival tissues.

## 5. Conclusions

Within the limitations of this study, while higher global DNA methylation levels in gingival tissue samples were found in comparison to bone, similar global DNA methylation levels were observed between the IF and PH groups overall. Yet, differences in the global DNA methylation levels between gingival tissues and bone could reflect a different epigenetic response between various tissues within the same microenvironment. Despite the limitations of this study, it still could serve as a first step for further investigations to analyze the DNA methylation of a specific gene panel related to inflammation and cytokine secretion, with an anticipated difference of the DNA methylation levels of this specific gene panel between gingival tissue and bone samples, based on the findings of the present pilot study. In fact, it would be interesting to unravel how the same gene is differentially methylated in the gingival tissue compared to bone.

In summary, further studies are necessary to elucidate the findings of the present study and to evaluate the association between epigenetic modifications and IF due to PIT.

## Figures and Tables

**Table 1:** Group comparison at baseline stratified by group (PH/IF).

		Healthy (PH)	Implant Failure (IF)	*p*-Value
**Number (n)**		10	9	
**Age (mean (SD))**		30.90 (6.76)	63.33 (11.84)	<0.001
**Gender (%)**	Female	7 (70.0)	6 (66.7)	1.000
	Male	3 (30.0)	3 (33.3)	
**Number of Dental Prophylaxis in the Past 2 Years (Median [IQR])**		2.00 [2.00, 2.00]	2.00 [2.00, 2.00]	0.478
**Number of Teeth (Including Prosthesis) (Median [IQR])**		32.00 [30.25, 32.00	24.00 [24.00, 28.00]	<0.001
**Number of Missing Teeth (Median [IQR])**		0.00 [0.00, 1.75]	23.00 [7.00, 32.00]	<0.001
**Number of Present Natural Teeth (Median [IQR])**		32.00 [30.25, 32.00]	9.00 [0.00, 24.00]	<0.001
**Number of Implants (Median [IQR])**		0.00 [0.00, 0.00]	8.00 [3.00, 11.00]	<0.001
**Number of Implants With PIT (Median [IQR])**		0.00 [0.00, 0.00]	2.00 [1.00, 4.00]	<0.001
**Periodontal Staging (%)**				<0.001
	Healthy	10 (100.0)	1 (11.1)	
	I-A	0 (0.0)	1 (11.1)	
	II-A	0 (0.0)	1 (11.1)	
	IV-A	0 (0.0)	1 (11.1)	
	IV-B	0 (0.0)	4 (44.4)	
	IV-No Teeth	0 (0.0)	1 (11.1)	

Abbreviations: IQR: interquartile range; PIT: peri-implantitis; SD: standard deviation.

**Table 2:** Baseline clinical parameters around failed dental implants.

	PD (mm)	BOP (%)	GR (mm)	KT (mm)	BL (mm)
Mean	9.22	89	1.17	2.89	5.77
SD	1.82	32	3.11	1.13	1.98

Abbreviations: BL: crestal bone loss; BOP: bleeding on probing; GR: gingival recession; KT: keratinized tissue height; PD: probing depth; SD: standard deviation.

**Table 3:** Intergroup comparison stratified by group.

	Healthy (PH)	Implant Failure (IF)	*p*-Value
**Bone and Gingival Tissue**			
Number (n)	20	18	
5-mC% (median [IQR])	2.48 [1.42, 5.40]	2.80 [1.98, 4.86]	0.599
**Only Bone**			
Number (n)	10	9	
5-mC% (median [IQR])	1.64 [0.43, 2.76]	2.08 [1.73, 2.63]	0.414
**Only Gingival Tissue**			
Number (n)	10	9	
5-mC% (median [IQR])	4.86 [2.33, 11.38]	5.01 [2.97, 10.50]	0.744

Abbreviations: 5-mC: global DNA methylation; IQR: interquartile range.

**Table 4:** Intragroup comparison stratified by the sample type.

	Bone	Gingival Tissue	*p*-Value
**Healthy (PH) and Implant Failure (IF)**			
Number (n)	19	19	
5-mC% (median [IQR])	1.76 [1.17, 2.85]	4.89 [2.60, 11.70]	0.001
**Only Healthy (PH)**			
Number (n)	10	10	
5-mC% (median [IQR])	1.64 [0.43, 2.76]	4.86 [2.33, 11.38]	0.019
**Only Implant Failure (IF)**			
Number (n)	9	9	
5-mC% (median [IQR])	2.08 [1.73, 2.63]	5.01 [2.97, 10.50]	0.009

Abbreviations: 5-mC: global DNA methylation; IQR: interquartile range.

## Data Availability

The data presented in this study are available on request from the corresponding author.

## Supplementary Materials

The following supporting information can be downloaded at: www.mdpi.com/article/10.3390/ijerph19021020/s1, File S1: Brief description of each recorded variable; Table S1: Implant characteristics.
