# Supplementary material for: Global DNA Methylation in Dental Implant Failure Due to Peri-Implantitis: An Exploratory Clinical Pilot Study
Source: Int J Environ Res Public Health. 2022 Jan 17;19(2):1020. doi: 10.3390/ijerph19021020 (PMC8775395; doi:10.3390/ijerph19021020)
Supplement: Supplementary file 1 [file ijerph-19-01020-s001.zip › ijerph-1523044-supplementary.pdf]

Global DNA Methylation in Dental Implant Failure due to Peri-implantitis: An Exploratory Clinical Pilot Study.

SUPPLEMENTARY I

BRIEF DESCRIPTION OF EACH RECORDED VARIABLE

Demographics

Subject demographics, including age, gender, race/ethnicity, and tobacco history were documented.

Medical and Dental History Recording

Relevant medical history (e.g., systemic diseases) and current medical conditions were recorded. The dental history included dental status information as well as a description of the dental implant characteristics.

Clinical Measurements

All clinical measurements were performed on teeth and dental implants at six locations; distobuccal (DB), buccal (B), mesiobuccal (MB), distopalatal (DP), palatal (P), mesiopalatal (MP) using a manual periodontal probe (UNC 15, Hu-Friedy, Chicago, IL, USA) by the same investigator. The following clinical measurements were evaluated:

1. Probing depth (PD): distance from the bottom of the pocket to the gingival margin (GM) in millimeters.
2. Gingival recession (GR): measurement of distance from Cemento Enamel Junction (CEJ) to GM.
3. Clinical attachment level (CAL): calculated by subtracting the GR from the PD.
4. Bleeding on probing (BOP): assessed after PD measurements. A dichotomous scoring system normally use with one (1) and zero (0) equaling presence or absence of bleeding 60 seconds after probing the pocket, respectively.
5. Keratinized tissue height (KT): assessed from free gingival margin to muco-gingival junction in mid-buccal for implants that were removed.

Radiographic Assessments

A full mouth radiograph series and/or a panoramic were done for patients that met the inclusion and to analyze their periodontal staging disease. In case of the IF group, an intra-oral radiograph and/or CBCT scan were performed for crestal bone loss (BL) analysis.

Table S1. Implant characteristics

	Subject ID		2	7	11	12	13	15	16	17	18
Implant Details	Implant Site (ADA)		13	31	20	31	13	5	29	19	29
	Manufacturer/System		Nobel Biocare	Nobel Biocare	BTI	Nobel Biocare	Klockner	BTI	BTI	Nobel Biocare	BTI
	Diameter		3,3	3,75	5	5	3,5	4	3,75	5	3,75
	Length		13	8,5	13	8,5	15	8,5	8,5	13	8,5
	Surface		MKIII TiUnite	NP TiUnite	Acid etched	MKIII TiUnite	Ti blasting	Optima	Acid etched	MKIII TiUnite	Acid etched
	Platform	Matching	✓	✓	✓	✓	✓	✓	✓	✓	✓
		Switching									
Prosthesis Details	Abutment	Stock	✓	✓	✓	✓	✓	✓	✓	✓	✓
		Custom									
	Restoration	Cemented				✓					
		Screw	✓	✓	✓		✓	✓	✓	✓	✓
	Loading	Immediate		unk	✓		unk	✓			
		Delayed	✓	unk		✓	unk		✓	✓	✓
	Type	Splint	✓	✓	✓	✓	✓	✓	✓		✓
		Non/Splint								✓	
Date of implant placement (years)			13.75	6.00	9.08	12.58	12.00	2.17	14.92	15.25	11.25
Date of implant loading (years)			13.58	unk	8.50	12.83	unk	2.17	14.58	15.00	10.75
